# The Many (Inter)faces of Anti-CRISPRs: Modulation of CRISPR-Cas Structure and Dynamics by Mechanistically Diverse Inhibitors

**DOI:** 10.3390/biom13020264

**Published:** 2023-01-31

**Authors:** Helen B. Belato, George P. Lisi

**Affiliations:** 1 Department of Molecular Biology, Cell Biology & Biochemistry, Brown University, Providence, RI 02903, USA; 2 Graduate Program in Therapeutic Sciences, Brown University, Providence, RI 02903, USA

**Keywords:** CRISPR-Cas9, anti-CRISPR, protein dynamics, allostery, inhibitor

## Abstract

The discovery of protein inhibitors of CRISPR-Cas systems, called anti-CRISPRs (Acrs), has enabled the development of highly controllable and precise CRISPR-Cas tools. Anti-CRISPRs share very little structural or sequential resemblance to each other or to other proteins, which raises intriguing questions regarding their modes of action. Many structure–function studies have shed light on the mechanism(s) of Acrs, which can act as orthosteric or allosteric inhibitors of CRISPR–Cas machinery, as well as enzymes that irreversibly modify CRISPR–Cas components. Only recently has the breadth of diversity of Acr structures and functions come to light, and this remains a rapidly evolving field. Here, we draw attention to a plethora of Acr mechanisms, with particular focus on how their action toward Cas proteins modulates conformation, dynamic (allosteric) signaling, nucleic acid binding, and cleavage ability.

## 1. Introduction

CRISPR-Cas systems are a form of bacterial adaptive immunity that consist of multiprotein complexes (called class 1) or single proteins (called class 2) with RNA-guided DNA endonuclease activity. CRISPR-Cas proteins protect bacterial cells from invasion by foreign pathogens (i.e., viruses, bacteriophages) by integrating short segments of the invasive genome into CRISPR loci, where it can template transcription of CRISPR RNA molecules. The ability of CRISPR-Cas to recognize and cleave DNA has been co-opted as a transformative laboratory tool, but its translational potential is hampered by deleterious off-target (or unexpected on-target) effects [1,2]. Cytotoxicity during in vitro and in vivo editing [3,4] and immunogenicity [5,6] must be addressed in further applications. Strategies to spatiotemporally control Cas nuclease activity are limited, but this technology would greatly benefit from engineered “on/off switches” that prevent unwanted editing. Current work has focused on limiting Cas nuclease activity via timed injection of preformed ribonucleoprotein (RNP) complexes [7,8,9] or the introduction of exogenous regulatory domains [10,11] and mutations [12,13] into Cas proteins, with varying degrees of success. Limitations of these approaches include substantial size increases for an already large Cas protein via appended regulatory domains or high-fidelity variants that are not universally effective. Temporal limitation of Cas-RNP activity is also difficult from a delivery standpoint (i.e., through a viral vector) and may require other exogenous stimuli to function in vivo.

In the same way that bacterial CRISPR-Cas technology has been adapted for bioengineering, an answer to enhanced spatiotemporal control of Cas systems may lie with the very pathogens that bombard this adaptive immune system. To escape CRISPR-mediated destruction, viruses and phage have evolved anti-CRISPR (Acr) proteins that suppress CRISPR-Cas function [14,15,16]. While many reviews comprehensively highlight the discovery, function, and evolution of Acrs [17,18], this review aims to emphasize the molecular mechanism of Acrs through a structural lens. Naturally occurring Acrs are small polypeptides (<150 amino acids) with unique structures, no significant sequence identity, and distinct mechanisms [17,19,20,21,22]. Acrs were initially discovered when researchers began comparing the genomes of bacteriophages that were affected by CRISPR-Cas9 systems with bacteriophages that were not [17]. More than 40 different Acrs have since been found to inhibit CRISPR-Cas function by interacting directly with a Cas protein to prevent target DNA binding or cleavage, guide RNA (gRNA) binding, or effector-complex formation.

Acr proteins are named based on the type of Cas system they target (AcrI for Class I, AcrII for Class II), their target subclass (i.e., Type-IIA, AcrIIA...), and their order of discovery (AcrIIA1, 2, 3…). To date, Acrs have been shown to regulate gene editing in several Cas proteins, most notably canonical Cas9s from *Streptococcus pyogenes* (*Sp*Cas9, i.e., AcrIIA2 and AcrIIA4) [23] and *Neisseria meningitidis* (*Nme*Cas9, i.e., AcrIIC1 and AcrIIC3) [24]. The controlled use of Acrs to “gate” Cas DNA editing is a tunable strategy to limit off-target effects without the need for specificity-enhancing mutations or fusion proteins. Acr genes can also be packaged into delivery vectors and deployed to maintain a dynamic range of Cas function, facilitating continuous protection of cells from editing, or fine tuning the duration of Cas activity [25]. AcrIIA4 has also been successfully introduced into CRISPR-based gene regulation circuits, where the down- or up-regulation of genes can be experimentally halted by the addition of AcrIIA4 in mammalian cells [25]. AcrIIA4 variants have also been engineered to become inactivated with light, followed by an assay that allows for temporal control of the CRISPR-Cas-Acr complex [26]. These systems can likely be developed for other Acr proteins to expand CRISPR-based biotechnology.

Many Acr proteins have also been found to inhibit multiple Cas orthologs [27,28,29,30], and broad-spectrum activity is advantageous in the regulation of CRISPR-Cas variants, eliminating the need to continuously re-engineer related Cas proteins. Despite this advantage, de novo prediction of novel Acrs is challenging due to the lack of a common structural link between them. An increasing number of studies related to the inhibitory underpinnings of Acr function highlight a subset of possible mechanisms that we will discuss using example case studies, focusing explicitly on structural and dynamic insight. When considered in totality, the molecular details of Cas-Acr complexes have the potential to inform strategies for structure-based design of new Acrs.

## 2. General Modes of Cas Inhibition by Acrs

The sequence and structural diversity of Acrs are reflected in mechanisms that include (1) inhibition via direct binding to and steric occlusion of the Cas functional sites, (2) modification of Cas conformational dynamics as nucleic acid mimics, (3) allosteric inhibition at regions distinct from the Cas functional sites, and (4) enzymatic degradation of the CRISPR–Cas components. Though the entire scope of Acr biochemistry is too great to cover in detail here, we will highlight Acr inhibitors that effectively bind to and prevent recruitment of Cas subunits to the Type-I Cascade–crRNA surveillance (Csy) complex or that interact directly with Csy to block DNA binding [31]. This CRISPR system is characterized by multiple Cas subunits coming together to form a complex around the CRISPR RNA (crRNA), with the Cas6f protein at the top of the complex, followed by six Cas7 subunits, a Cas5 subunit, and a Cas8f subunit. The Cas subunits and crRNA work together as a complex to recognize and cleave target DNA. We will also detail examples of Acrs that target Type-II Cas systems to interfere with nucleic acid recognition and binding [32,33,34,35,36,37], gRNA loading [33], or DNA cleavage via the nucleases [36]. We will discuss critical examples of each inhibitory mechanism, focusing on the structural insight gleaned from the case studies.

## 3. Direct Interaction of Acrs with Cas Nucleases (HNH or RuvC)

**Figure 1:**
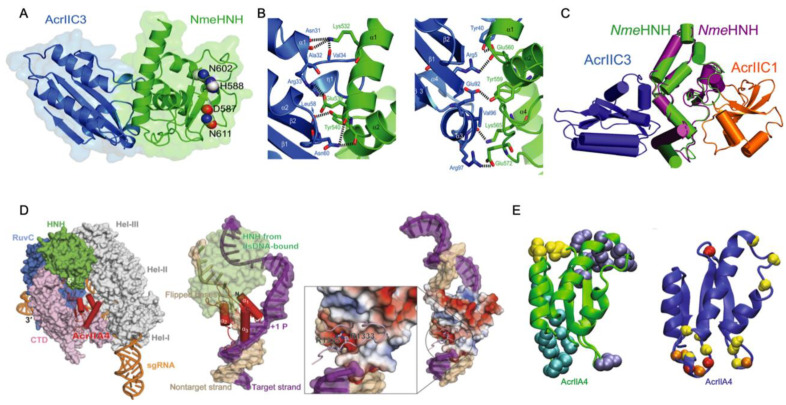
Direct interaction of Acrs with Cas nucleases. (**A**) Structure of AcrIIC3 (blue) bound to *Nme*HNH (green) at an interface opposite the active site pocket (residues shown as spheres). (**B**) Interaction interface between AcrIIC3 (blue) and *Nme*HNH (green) showing intermolecular hydrogen bonds and salt bridges. (**C**) Demonstration of a plausible dual-Acr ternary complex via the structure of AcrIIC3-*Nme*HNH (blue, green) superimposed onto the structure of AcrIIC1-*Nme*HNH (orange, purple, PDB 5VGB). (**D**) Superposition of the AcrIIA4-bound *Sp*Cas9 structure with DNA-bound *Sp*Cas9 showing AcrIIA4 blocking DNA recognition at the PAM binding pocket (left). Superposition of the AcrIIA4-bound *Sp*Cas9 structure with *Sp*Cas9-gRNA-dsDNA (PDB 5F9R) showing PAM recognition residues buried in an AcrIIA4 acidic pocket (middle, right). (**E**) Binding interfaces of AcrIIA4 to *Sp*Cas9 RNP are shown as spheres (PDB ID: 5VW1). Yellow, cyan, and dark blue colored residues correspond to binding to the *Sp*Cas9 topoisomerase-homology domain, RuvC domain, and C-terminal domain, respectively (left). Residues of AcrIIA4 with conformational flexibility are highlighted as red spheres (fast ps-ns timescale), yellow spheres (slow µs-ms timescale), or orange spheres (both fast and slower motions). Figure 1A–C adapted with permission from Kim, Y. et al. Anti-CRISPR AcrIIC3 discriminates between Cas9 orthologs via targeting the variable surface of the HNH nuclease domain [36]. Figure 1D is adapted from Shin, J. et al. Disabling Cas9 with an anti-CRISPR DNA mimic [40]. Figure 1E is adapted from Kim, I. et al. Solution structure and dynamics of anti-CRISPR AcrIIA4, the Cas9 inhibitor [31].

Acrs targeting the catalytic centers of Cas proteins have been reported as potent temporal and spatial regulators of function. All Type-II Cas9 proteins harbor two nuclease domains: an HNH-like domain (named for its catalytic His and Asn residues) and the RuvC-like domain, which work together to cleave target DNA that is complimentary and non-complimentary to the gRNA, respectively. The Acr-Cas pair is somewhat organism-specific, but broad-spectrum effects of Acrs have been noted, particularly in cases where the conserved catalytic residues of the Cas nucleases are targeted. Recently, a Type II-C anti-CRISPR, AcrIIC1, was shown to bind directly to the *Nme*Cas9 HNH domain and prevent target DNA cleavage. Structural studies of AcrIIC1 by Suh and coworkers demonstrated a direct interaction with the active site residues of *Nme*HNH, thereby trapping the target-bound effector complex in a catalytically inactive state [37]. In the same study, a related Acr, AcrIIC3, also targeted *Nme*HNH, but at a different binding interface, as demonstrated through the crystal structure of the AcrIIC3–HNH complex with AcrIIC3 bound opposite the *Nme*HNH active site (Figure 1A,B). This novel inhibitory interface suggests that AcrIIC3 discriminates between Cas9 orthologs, binding solely to *Nme*HNH in contrast to the broad-spectrum inhibition of AcrIIC1 and other Acrs targeting the conserved catalytic pocket [27,38,39]. In fact, *Nme*AcrIIC1 also exhibits strong inhibition against *Geobacillus stearothermophilus* Cas9 (*Geo*Cas9) and *Campylobacter jejuni* Cas9 (*Cje*Cas9) [27]. However, the interaction surface for AcrIIC3 on *Nme*HNH is highly variable in Cas9 orthologs. For example, contacts between AcrIIC3 and *Nme*HNH are facilitated by Lys532, Glu560, and Glu572 of *Nme*HNH, among other residues, which participate in crucial hydrogen bonds and salt bridges with AcrIIC3. Although *Nme*HNH, *Geo*HNH, and *Cje*HNH are structurally homologous, *Geo*HNH and *Cje*HNH possess chemically divergent amino acids in these positions. Suh and coworkers mutated these three key residues in *Nme*HNH to match those of *Cje*HNH, which dramatically decreased the AcrIIC3 binding affinity to the *Cje*HNH mimic. The same effect was observed with a mutationally constructed *Geo*HNH mimic, suggesting that AcrIIC3 is tuned to specifically target the *Nme*HNH nuclease.

Interestingly, the crystal structures of *Nme*HNH-AcrIIC3 appear as a dimer, which was previously suggested as the mechanism by which AcrIIC3 disrupts target DNA binding of NmeCas9. However, the oligomeric state of AcrIIC3 was shown to be monomeric in chromatograms by Suh and coworkers, suggesting a crystal packing artefact. *Nme*Cas9-gRNA in complex with DNA does in fact dimerize when bound to AcrIIC3, as evidenced by crystal structures of *Nme*Cas9-gRNA-DNA-AcrIIC3 that show an AcrIIC3 binding interface with an additional interaction at the nucleic acid recognition (REC) lobe of another *Nme*Cas9 complex (also see *Acr-Driven Oligomerization of Cas Proteins* below). Crystal structure superimpositions of *Sp*Cas9-gRNA (PDB 4ZT0) [41], *Sp*Cas9-gRNA-PAM duplex (a partially duplexed DNA with a PAM-containing segment; PDB 4UN3) [42], and *Sp*Cas9-gRNA-DNA (PDB 5F9R) [43] with *Nme*HNH-AcrIIC3 show that while the *Sp*Cas9–gRNA complex accommodates AcrIIC3 without any steric hinderance, its gRNA-DNA-bound complexes cannot bind AcrIIC3 without severe overlap between the REC lobe of *Sp*Cas9 and AcrIIC3. Thus, AcrIIC3 discriminates between Cas9 orthologs via sterics at a variable surface of HNH. The specific binding mode of AcrIIC3 to *Nme*HNH suggests, remarkably, that *Nme*HNH, AcrIIC3, and AcrIIC1 can form a ternary complex without any steric clash. (Figure 1C).

Similarly, AcrIIA4, which is comprised of three antiparallel β-strands flanked by three α-helices, inhibits *Sp*Cas9 nuclease function, though structures of the AcrIIA4–*Sp*Cas9–gRNA complex obtained by X-ray crystallography and electron microscopy show that *Sp*Cas9 binds to AcrIIA4 via the protospacer adjacent motif (PAM) interaction site and adjacent RuvC nuclease rather than HNH (Figure 1D,E) [40,44,45]. The inhibitory activity of AcrIIA4 was attributed by Kim and coworkers to be due to extensive micro-millisecond backbone dynamics on multiple timescales in loops that form interaction surfaces with *Sp*Cas9, based on ^15^N NMR spin relaxation experiments. In addition to slower dynamics in the loops joining AcrIIA4 secondary structure elements, several loop residues within AcrIIA4 appeared severely line broadened, consistent with increased transverse relaxation due to conformational dynamics on the micro-millisecond timescale. Interestingly, the region of direct interaction with RuvC appears highly flexible with fast (pico-nanosecond) internal motions, as measured by heteronuclear NOEs. This study revealed how the distinct dynamic regimes of AcrIIA4 not only transmit chemical information to critical structural elements of Cas9 but also organize the Acr-Cas binding interface in a way that is critical to its mechanism of inactivation.

The ability of Acrs to inhibit Cas9 function by binding to its functional sites is a conserved strategy among Acrs that target Type-II Cas systems. However, the Acrs discussed as case studies clearly utilize different mechanisms. AcrIIC1 is a broad-spectrum Cas9 inhibitor that directly binds to the active site of the HNH domain. AcrIIC3 selectively inhibits *Nme*Cas9 via hydrogen bonding networks, salt bridge networks, and hydrophobic interactions with HNH. AcrIIA4 has a highly dynamic binding interface with the PAM-Interacting and RuvC domains of *Sp*Cas9 in the absence of gRNA, where the *Sp*Cas9-AcrIIA4 complex can still bind gRNA but not target DNA. Given the array of possible mechanisms, understanding the critical structural elements for Acr-Cas binding and subsequent inhibition is necessary to guide future efforts to fine-tune Cas function with designed Acrs.

## 4. Steric Occlusion of Nucleic Acids by Acrs

Anti-CRISPRs most commonly block access to the functional sites of Cas proteins; however, an equally effective mode of steric control is achieved at the gRNA, or target DNA, level. For example, AcrIF10 acts as a DNA mimic to prevent activation of the Type-I Csy complex, while AcrIIA2 and AcIIA4 play similar roles in Type-II Cas9-gRNA complexes. These and other nucleic acid-mimicking Acrs can (1) bind at the junction between Cas subunits to induce a DNA-bound conformation [46], (2) interact with PAM binding elements to prevent the recognition of the PAM adjacent to the target sequence [40,44,45,47,48], or (3) induce conformational changes that preclude target DNA binding [46].

**Figure 2:**
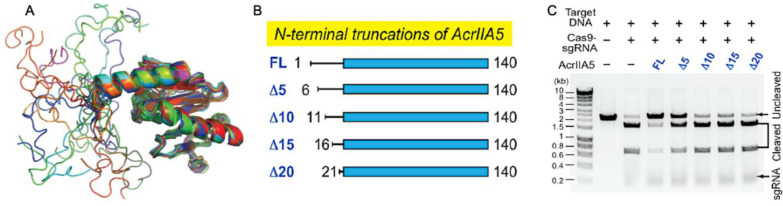
Steric occlusion of nucleic acids by Acrs. (**A**) Superimposed ensemble of 20 NMR structures of AcrIIA5 demonstrating N-terminal disorder preceding a highly structured domain. (**B**) Engineered constructs of AcrIIA5 with N-terminal IDR truncations. (**C**) Functional impact of IDR truncations on the inhibitory activity of AcrIIA5 against Cas9. DNA cleavage assays demonstrate that AcrIIA5 is incapable of arresting Cas9 cleavage function in the absence of its N-terminal IDR. Figure 2A–C is reprinted/adapted from An, S. Y. et al. Intrinsic disorder is essential for Cas9 inhibition of anti-CRISPR AcrIIA5 [34].

Focusing on the Acr structure and building on the conformational dynamics highlighted in Acr-Cas nuclease interactions, An and coworkers introduced a role for intrinsic disorder in an Acr targeting the gRNA binding cleft. A solution structure of AcrIIA5 featured an N-terminal intrinsically disordered region (IDR), a first among Acrs, and biophysical studies proved that truncation of the IDR modulated both the association between AcrIIA5 and Cas9–gRNA and the catalytic efficiency of the inhibitory complex in vitro [34]. When its 20 N-terminal IDR residues were removed, AcrIIA5 maintained the identical backbone fold within its structured region, as evidenced by highly similar ^1^H–^15^N NMR chemical shifts, but completely lost its ability to inhibit Cas9, revealing that the N-terminal IDR was essential for Acr activity (Figure 2). The N-terminal IDR of AcrIIA5 is rich with basic (positively charged) lysine and arginine residues, and upon neutralization of these charges via alanine mutations, inhibitory activity against Cas9 was diminished following a trend of R12A/K13A/R14A > K5A/R7A ≈ R18A/K21A. This suggests that the positive charges in the IDR of AcrIIA5 are critical for interaction with and inhibition of Cas9. The charge content of the IDR was strongly correlated to AcrIIA5 inhibitory activity, while the length of the IDR affected the Acr-Cas interaction in electrophoretic mobility shift assays (EMSA). EMSA clearly demonstrated a ternary complex between Cas9, gRNA, and AcrIIA5 upon titration of AcrIIA5 into the Cas9-gRNA complex, and IDR truncations gradually attenuated the strength of the interaction. Concomitant NMR experiments revealed significant line broadening of AcrIIA5 amide resonances in the presence of Cas9-gRNA, but only in the presence of the N-terminal IDR and contingent on a preformed Cas9-gRNA complex.

**Figure 3:**
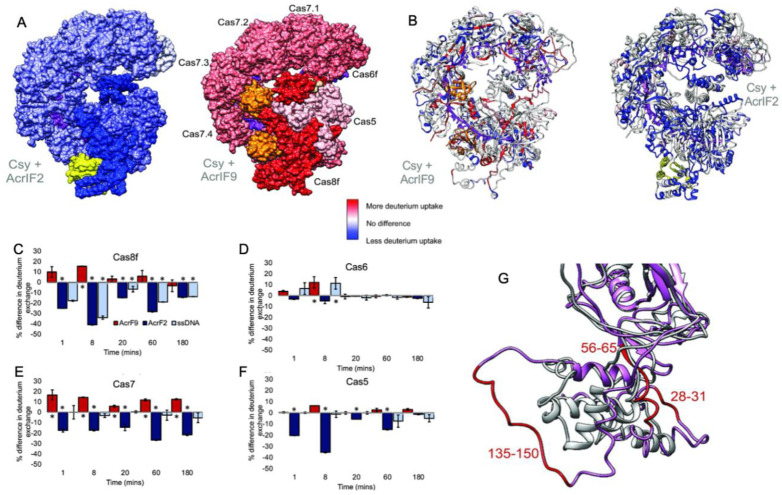
Dynamics of the multi-Cas Csy complex induced by Acrs. (**A**) Intact HXD map of total deuterium exchange in the Csy complex bound to AcrIF2 (left) and AcrIF9 (right), relative to the apo Csy complex. Structures are heat mapped according to the legend. AcrIF2 is shown in yellow, AcrIF9 in orange, and crRNA is shown in purple. (**B**) Peptide level HDX monitoring specific regions within the Cas subunits affected by Acrs. Structures are heat mapped according to the same legend. Generally, strong dynamic perturbations to the Csy complex occur proximal to the AcrIF2/9 binding sites. (**C**–**F**) Differences in deuterium uptake for various Csy subunits in the presence of AcrIF2 (dark blue), AcrIF9 (red), or ssDNA (light blue), compared to the apo Csy complex. Positive values reflect increased deuterium uptake via enhanced protein dynamics. (**G**) Structural dynamics of the Cas8 subunit of Csy captured in the presence of AcrIF9. Comparison of the N-terminal region (1–166) of apo Cas8/Csy (gray) and AcrIF9-bound Cas8/Csy (pink) shows several peptides with altered structures, reflective of enhanced deuterium uptake. Figure reprinted/adapted from Patterson, A. et al. Anti-CRISPR proteins function through thermodynamic tuning and allosteric regulation of CRISPR RNA-guided surveillance complex [32]. * indicates a significant *p*-value < 0.05.

In a related study, hydrogen-deuterium exchange-mass spectrometry (HDX) explored the binding of two different Acrs (AcrIF2 and AcrIF9) to the large Type-I CRISPR surveillance complex (Csy). Here, Patterson and coworkers demonstrated dynamic contributions to Acr binding via enthalpic (AcrIF2) and entropic (AcrIF9) changes to the Csy conformational landscape [31]. Though both Acrs interfere with Cas nucleic acids, distinct subunits of the multi-Cas complex are affected by the Acrs, identified through subtle conformational changes occurring distal to the Acr binding site that hint at long-range allostery in Acr inhibition of Csy. AcrIF2 shares a similar electrostatic surface potential to DNA and stabilizes Csy [49], which was confirmed by significantly less deuterium uptake (relative to apo Csy) localized to the Cas7/Cas8 interface in HDX studies (Figure 3). The reduction in HDX suggests a rigidification of the Csy structure via enthalpic stabilization, consistent with changes that would be expected of DNA. In contrast, AcrIF9-bound Csy displayed significantly increased deuterium uptake along the gRNA interface, which is proximal to AcrIIF9, as well as in regions more than 30 Å from the gRNA. The AcrIIF9 binding site is also distinct from that of AcrIIF2, which is found distal to the gRNA. Two AcrIF9 molecules are seen bound to Cas7 subunits of Csy in an orientation that sterically hinders binding of the target DNA to the gRNA. Increased deuterium uptake in this structure indicates the Cas7 subunits become more flexible in the presence of AcrIF9 via an entropically dominate effect. Large-scale changes to the HDX profile of Csy in the presence of either AcrIF2 or AcrIF9 demonstrate how the hydrogen bonding network of each complex is remodeled with thermodynamically opposing driving forces. Differences in the HDX profiles between apo-Csy and the Csy-Acr complex highlight unique conformational states sampled by each system. The ensemble of structures populated by Csy is dependent on the energetics of those states, and the binding of Acrs alters the conformational freedom of Csy, which appears to be increased with AcrIF9 bound and decreased with AcrIF2 bound. Acr binding to Csy affects HDX not only at the binding interface but also in distant sites on the protein complex, suggesting an allosteric network within Csy, consistent with other Type-I CRISPR systems.

## 5. Interaction with the Nucleic Acid Bridge Helix

**Figure 4:**
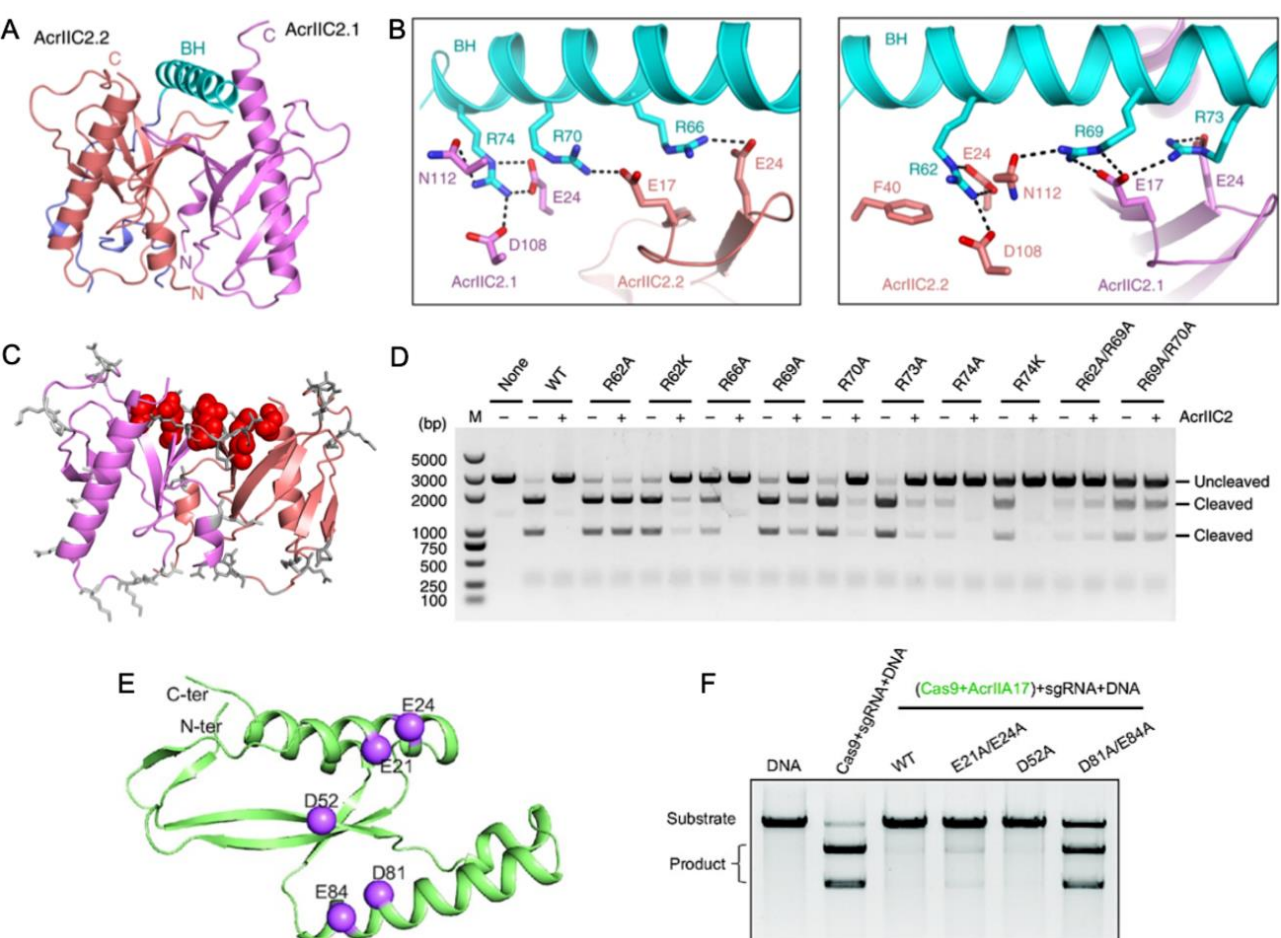
Acr interactions with the nucleic acid bridge helix (**A**) Crystal structure of AcrIIC2 depicts a homodimer (red and purple subunits) that interacts with the *Nme*Cas9 bridge helix (cyan). (**B**) Hydrogen bond and salt bridge interactions between the *Nme*Cas9 bridge helix (cyan) and identical subunits of AcrIIC2 (red and purple). (**C**) Summary of surface-exposed residues hypothesized to have functional impact on the AcrIIC2-*Nme*Cas9 interaction. Sites with no functional impact when mutated are depicted as gray sticks, while those that strongly affect the ability of AcrIIC2 to inhibit *Nme*Cas9 are shown in red. (**D**) DNA cleavage assay conducted in the presence of *Nme*Cas9 and wild-type AcrIIC2, as well as AcrIIC2 variants outlined in (**C**). Mutations along the bridge helix-interacting region of AcrIIC2 arrest its inhibitory function. (**E**) Predicted AlphaFold structure of AcrIIA17 showing hypothesized sites of functional importance based on charge complementarity to the Cas9 surface (purple spheres). (**F**) Alanine mutations at the highlighted sites in (**B**) attenuate the affinity of AcrIIA17 for Cas9 and modulate its ability to inhibit Cas9 DNA cleavage. Figure 4A–D is reprinted/adapted from Thavalingam, A. et al. Inhibition of CRISPR-Cas9 ribonucleoprotein complex assembly by anti-CRISPR AcrIIC2 [33]. Figure 4E,F is reprinted/adapted from Wang, X. et al. Inhibition mechanisms of CRISPR-Cas9 by AcrIIA17 and AcrIIA18 [39].

Another way in which Acrs can mimic Cas nucleic acids for inhibition is in the targeting of the bridge helix connecting the recognition (REC) and nuclease (NUC) lobes of Cas9. Within the NUC lobe is the PAM-interacting domain, which is variable in structure and often disordered in the absence of nucleic acids [50]. The transition of Cas9, for example, to its active conformation requires gRNA binding mediated by the arginine-rich bridge helix [51], followed by substantial structural rearrangements in the REC lobe [41]. Thavalingam and coworkers investigated the recognition of AcrIIC2 by the bridge helix of *Nme*Cas9, first demonstrating that AcrIIC2 co-eluted with the REC lobe during chromatographic purification [33]. Constructs of *Nme*Cas9 lacking the bridge helix (REC-ΔBH) were unable to co-elute with AcrIIC2. Further confirmation of its critical role in AcrIIC2 recognition was provided through REC subdomain deletion constructs that maintained interaction with AcrIIC2 via the bridge helix despite losing other regions of the REC structure. A 2.5 Å crystal structure of AcrIIC2 bound to *Nme*Cas9 further informs the mechanism, which depicts AcrIIC2 as a dimer in complex with only residues 16–77 of *Nme*Cas9, corresponding to its bridge helix and a small portion of the adjacent RuvC nuclease. (Figure 4A,B). The AcrIIC2 dimer presents a negatively charged surface via four residues from each of the AcrIIC2 monomers, E17, E24, D108, and N112, that make interactions with the positively charged bridge helix. Mutations E17A, E24A, and D108A completely disrupted Acr activity in vivo, while mutations of non-specific residues distributed widely across the surface of AcrIIC2 showed changes in Cas9 activity of less than ten percent (Figure 4C,D). This indicates that in the case of AcrIIC2, its negatively charged surface comprises the critical interaction interface for the positively charged *Nme*Cas9 bridge helix and is required for CRISPR inhibition. The necessity of this charged interaction was reciprocated through neutralizing Ala mutations within the bridge helix itself, which also decreased the inhibition of *Nme*Cas9 by AcrIIC2. In contrast, substitutions with Lys, which maintain the same charge, retain the inhibitory effect.

Wang and coworkers reported that AcrIIA17 also engages with the *Sp*Cas9 bridge helix to inhibit RNP complex assembly [52]. The binding affinity of Acrs for Cas proteins is variable, and AcrIIA17 was found to weakly associate with *Sp*Cas9 based on poor co-migration in gel-filtration chromatography experiments. Interestingly, AcrIIA17 was shown in the same study to bind the related *Nme*Cas9 much more tightly, based on stable co-elution via gel filtration. The AlphaFold2 model of AcrIIA17 illustrates a negatively charged surface as the potential binding interface of the positively charged bridge helix of Cas9 (Figure 4E). In vitro DNA cleavage assays established that Cas9 activity is inhibited by AcrIIA17 only when AcrIIA17 is bound prior to Cas9-RNP formation, and the addition of AcrIIA17 after RNP formation does not inhibit Cas9 (Figure 4F). Therefore, AcrIIA17 competes with the gRNA for the bridge helix to impair RNP formation, effectively turning off the Cas9 function.

## 6. Acr-Driven Oligomerization of Cas Proteins

While broad-spectrum Acrs targeting conserved catalytic pockets of electrostatic surfaces have expanded our understanding of Acr function, recent reports of allosteric Acrs present an avenue toward greater customization and spatiotemporal regulation of Cas systems in vitro and in vivo. Allosteric Acrs are more likely to be organism-specific but may provide clues about underexplored Cas structural elements that can be targeted with de novo-designed Acrs. Recently, the AcrIIA6, AcrIIC3, and AcrVA4 proteins were shown to allosterically inhibit the *Streptococcus thermophilus* CRISPR1-Cas9 (*St1*Cas9), *Nme*Cas9, and *Lachnospiraceae bacterium* Cas12a (*Lb*Cas12a) complexes, respectively. In each case, the allosteric pocket for these Acrs allowed for the binding and inactivation of two Cas proteins at once. AcrIIA6 and AcrVA4 are both dimers that recognize protein-gRNA interfaces, as confirmed by recent cryo-EM structures [35].

**Figure 5:**
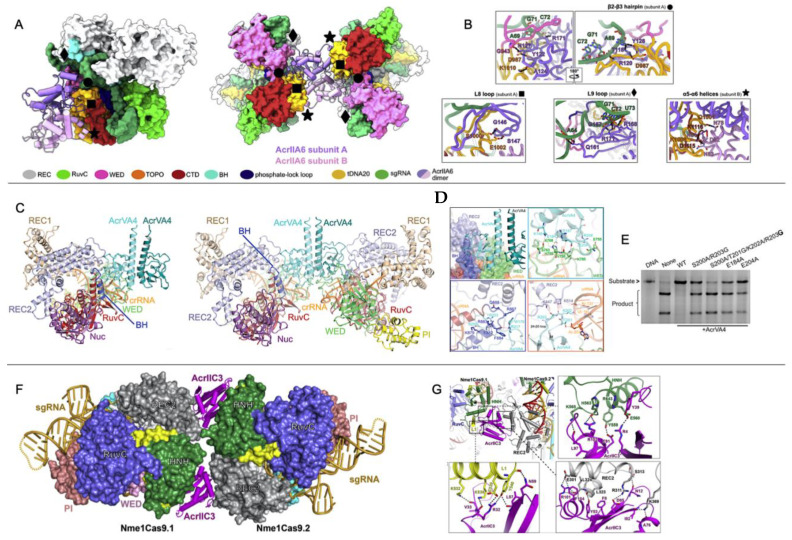
Acr-driven oligomerization of Cas proteins (**A**) Domain-specific interactions of the AcrIIA6 dimer (purple/pink) with *St1*Cas9. AcrIIA6 allosterically inhibits the *St1*Cas9 monomer (left) or dimer (right) via intermolecular interactions at sites noted by black diamonds, circles, squares, and stars. (**B**) Close-up views of the AcrIIA6 interactions at selected sites within the *St1*Cas9-sgRNA complex. (**C**) Cryo-EM structures of AcrVA4 dimer in complex with one copy (left) or two copies (right) of *Lb*Cas12a-crRNA. (**D**) Close up views on AcrVA4 interactions with REC2, WED domain, the bridge helix (BH), and the crRNA. (**E**) An in vitro DNA cleavage assay of *Fb*Cas12a in the presence of wild-type or mutant AcrVA4 highlights the inability of AcrVA4 to inhibit *Lb*Cas12a function when BH and cRNA binding residues are mutated. (**F**) Crystal structure of *Nme*Cas9-gRNA-AcrIIC3 complex showing two *Nme*Cas9-gRNA complexes bound to one AcrIIC3. (**G**) Close-up views of the interactions between AcrIIC3 and *Nme*HNH, *Nme*Rec2, and the L1 linker of *Nme*Cas9. Figure 5A,B is reprinted/adapted from Fuchsbauer, O. et al. Cas9 allosteric inhibition by the anti-CRISPR protein AcrIIA6 [35]. Figure 5C–E reprinted/adapted from Zhang, H. et al. Structural basis for the inhibition of CRISPR-Cas12a by anti-CRISPR proteins [37]. Figure 5F,G is reprinted/adapted from Sun, W. et al. Structures of *Neisseria meningitidis* Cas9 complexes in catalytically poised and anti-CRISPR-inhibited states [59].

The mechanism of Cas9 inhibition by AcrIIA6 is to disrupt *St1*Cas9 conformational dynamics that normally work to assist PAM binding and trigger large reorientations of the HNH and RuvC nucleases to achieve the active state [53,54,55,56]. Interestingly, two structural populations of the AcrIIA6-*St1*Cas9 complex can be observed by cryo-EM: a major particle class (~75%) showing a canonical *St1*Cas9 monomer and a minor class (~25%) with a symmetric, elongated *St1*Cas9 dimer. AcrIIA6 is observed to occupy a large surface of the *St1*Cas9-gRNA complex, but in a region distinct from the DNA-binding cavity or the catalytic sites. The AcrIIA6 binding interface is also identical in the monomeric and dimeric assemblies and is driven by extensive van der Waals contacts, a buried hydrophobic surface area of ~2300 Å^2^, and polar contacts from the AcrIIA6 β2-β3 hairpin (residues 119–133), L8 loop (142–148), and L9 loop (156–171) [35], as seen in Figure 5A,B. AcrIIA6 is not structurally perturbed in its complex with *St1*Cas9-gRNA, and based on its position in an allosteric site proximal to the PAM binding site, AcrIIA6 is primed to act on *St1*Cas9 either by inhibition of DNA binding or inhibition of nuclease activity. Fuchsbauer and coworkers noted the effect of AcrIIA6 to be very subtle, as PAM binding precipitates structural rearrangements within the PAM-interacting domain, which is displaced 1.8 Å from the PAM duplex, which is also shifted ~2.0 Å by AcrIIA6. These AcrIIA6-induced motions within *St1*Cas9 are consistent with an allosteric linkage between the AcrIIA6 binding and the *St1*Cas9 nucleic acid sites that constrains the architecture of the PAM-Interacting domain in such a way that it precludes target DNA binding and cleavage. AcrIIA6 is distinct in its mechanism in that it (1) does not sterically occlude the DNA binding site, and (2) does not prevent PAM recognition, in contrast to AcrIIA4 and AcrIIA2. In fact, AcrIIA6 binds to *St1*Cas9-gRNA-DNA assemblies, but with a lower affinity [35]. In a *St1*Cas9-gRNA-DNA-AcrIIA6 complex with a non-target PAM, the PAM-Interacting domain, which comprises the main AcrIIA6 binding interface, has already adopted an energetically favorable position to accommodate DNA, which explains the slower association and a faster dissociation rate of AcrIIA6 in these complexes compared to *St1*Cas9-gRNA as measured in biolayer interferometry.

In a Cas12a system, AcrVA4 exists as a dimer where each monomer can bind a *Lb*Cas12a-crRNA complex (from *Lachnospiraceae bacterium ND2006*). One or two *Lb*Cas12a-crRNA complexes can be bound to the AcrVA4 dimer (Figure 5C), and cryo-EM and gel filtration chromatography suggest that both species are in a dynamic equilibrium [57]. AcrVA4 binds with sub-nanomolar affinity due to the extensive interactions with *Lb*Cas12a driven by salt bridges, hydrophobic interactions, and cation-pi stacking interactions with REC2, the wedge (WED) domain, which is involved in recognition of the gRNA, the BH motif between the NUC and REC lobes, and the crRNA (Figure 5D). Cryo-EM highlights conformational changes of *Lb*Cas12a caused by crRNA association that creates a binding pocket of AcrVA4, consistent with previous observations that AcrAV4 binds to *Lb*Cas12a in a crRNA-dependent way. Zhang and coworkers demonstrated the importance of the interactions between AcrVA4 and the BH/crRNA b4-b5 loop by introducing mutations in AcrVA4 that disrupted these connections and impaired AcrVA4 inhibition of *Lb*Cas12a (Figure 5E) [37]. It is thought that AcrVA4 arrests the dynamics of the *Lb*Cas12a-crRNA complex such that the crRNA is unable to hybridize the target DNA.

In another case, a single AcrIIC3 monomer can tether two *Nme*Cas9-gRNA complexes. As previously discussed, AcrIIC3 binds *Nme*HNH opposite the catalytic site but can also dock at the REC lobe of another *Nme*Cas9 complex (Figure 5F,G) [36,58,59]. The binding interfaces of AcrIIC3 and *Nme*REC are adjacent to the gRNA-DNA hybridization site, likely allosterically modulating the structure and dynamics of the REC lobe that position HNH in its active state [54,60]. NmeCas9-gRNA-AcrIIC3 complexes cannot bind target DNA in cells [24,27] and therefore must be locked in inactive conformations [59].

## 7. Enzymatic Modification of Cas9 Nucleic Acids by Acrs

Comparatively few Acrs have been shown to possess enzymatic activity against Cas systems. In addition to the Acrs that cleave gRNA at multiple sites in the spacer sequence, two related Acrs, AcrVA1 and AcrVA5, were shown by Knott and coworkers to inhibit Cas12a through enzymatic function. Interestingly, AcrVA1 and AcrVA5 bind to overlapping regions in the PAM-interacting domain and compete with one another for the binding site, though each Acr possesses distinct substrate specificity and enzymatic activity [21,37]. AcrVA1 is a multiple-turnover endoribonuclease that cleaves the Cas12a-crRNA spacer sequence to irreversibly inactivate the complex [21], by mimicking the PAM to position its catalytic residues in proximity to the gRNA [37]. Cas12a has also been shown to undergo lysine acetylation via AcrVA5 coupled to an acetyl-CoA cofactor, suggesting a suite of Acrs may possess wide-ranging capabilities to chemically modify Cas proteins [22].

**Figure 6:**
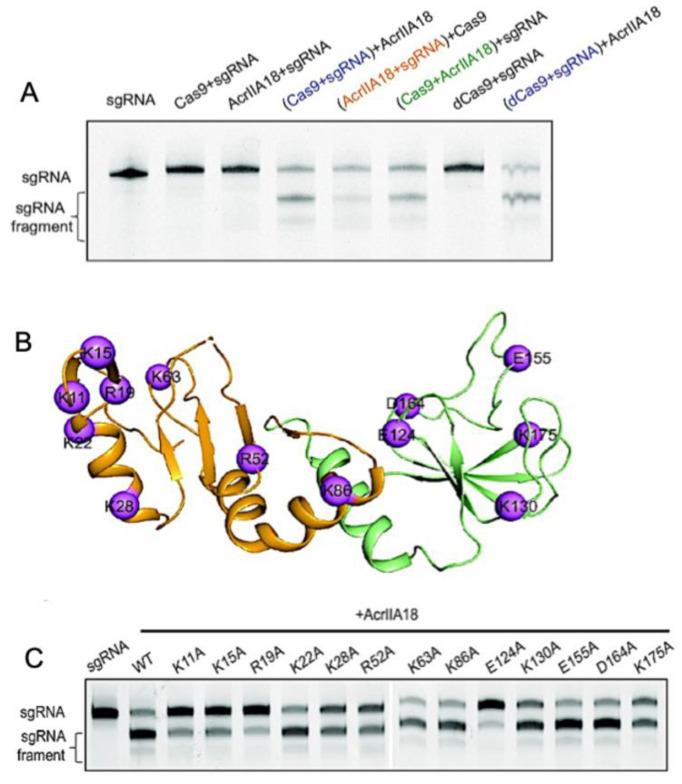
Enzymatic modification of Cas9 nucleic acids by Acrs. (**A**) Denaturing gel demonstrating that AcrIIA18 digests gRNA in a Cas9-dependent manner. (**B**) Sites of hypothesized functional importance in AcrIIA18 based on charge complementarity to Cas9 (purple spheres). (**C**) Alanine mutations at the highlighted sites in (**B**) modulate the ability of AcrIIA18 to enzymatically cleave gRNA. Figure is reprinted/adapted from Wang, X. et al. Inhibition mechanisms of CRISPR-Cas9 by AcrIIA17 and AcrIIA18 [39].

In Type-II Cas9s, AcrIIA18 catalyzes the truncation of gRNA, generating a guide that is incapable of activating the protein [52]. AcrIIA18 alone showed no obvious RNA degradation activity; however, in the presence of *Sp*Cas9, AcrIIA18 degraded gRNA, leaving only a short 15 nt spacer that is not long enough to activate *Sp*Cas9. Wang and coworkers rightfully hypothesized that the charged solvent-exposed residues of AcrIIA18 were functionally critical to the binding of and catalysis toward negatively charged gRNA. Neutralizing point mutations throughout the AcrIIA18 structure restored *Sp*Cas9 activity and highlighted key residues necessary for Cas9 inhibition by AcrIIA18. Catalytic residues with RNase activity cluster in a V-shaped groove on the N-terminal β-hairpin of AcrIIA18 (Figure 6), suggesting a critical role for structural complementarity with the *Sp*Cas9 nucleic acid binding cleft.

## 8. Conclusions

It is recognized that Acrs inhibit Cas proteins by highly varied mechanisms, likely due to the divergent evolution of small Acr proteins. However, the structural underpinnings of these processes are not always well understood. Exploring the biophysical principles important for Acr function is essential to pinpointing the most common architectural or dynamic features of Acr-Cas interactions, which can be used to predict future inhibitory outcomes with novel or designed Acrs.
